# A Comprehensive Kinetic Study on the Enhanced Thermal Stability of Silica Xerogels with the Addition of Organochlorinated Substituents

**DOI:** 10.3390/gels12010002

**Published:** 2025-12-19

**Authors:** Beatriz Rosales-Reina, Guillermo Cruz-Quesada, Pablo Pujol, Santiago Reinoso, César Elosúa, Gurutze Arzamendi, María Victoria López-Ramón, Julián J. Garrido

**Affiliations:** 1 Departamento de Ciencias, Institute for Advanced Materials and Mathematics (INAMAT^2^), Universidad Pública de Navarra (UPNA), Campus de Arrosadía, 31006 Pamplona, Spain; beatriz.rosales@unavarra.es (B.R.-R.); santiago.reinoso@unavarra.es (S.R.); 2 Departamento de Química Inorgánica y Orgánica, Facultad de Ciencias Experimentales, Universidad de Jaén, 23071 Jaén, Spain; gcruz@ujaen.es (G.C.-Q.); mvlro@ujaen.es (M.V.L.-R.); 3 Unidad Científico Técnica de Apoyo a la Investigación (UCTAI), Universidad Pública de Navarra (UPNA), Campus de Arrosadía, 31006 Pamplona, Spain; 4 Departamento de Ingeniería Eléctrica, Electrónica y de Comunicación, Institute of Smart Cities (ISC), Universidad Pública de Navarra (UPNA), Campus de Arrosadía, 31006 Pamplona, Spain; cesar.elosua@unavarra.es

**Keywords:** hybrid silica xerogel thermal stability, kinetic analysis, FWO method, Criado master plot

## Abstract

Hybrid silica xerogels functionalised with chlorinated organosilanes combine tunable porosity and surface chemistry, rendering them attractive for applications in sensing, membrane technology, and photonics. This study quantitatively investigates the thermal decomposition kinetics of organochlorinated xerogels and correlates with volatile compounds previously identified via Thermogravimetric Analysis (TGA) coupled to Fourier-Transform Infrared Spectroscopy (FT–IR) and Gas Chromatography coupled with Mass Spectrometry (GC–MS). The xerogels were synthesised via the sol–gel process using organochlorinated alkoxysilane precursors and yielded highly condensed nanostructures in which the precursor nature strongly influences the morphology and textural properties. In this study, the molar percentage of the organochlorinated compounds was fixed at 10%. Standard N_2_ adsorption-desorption isotherm at 77 K revealed that increasing the precursor content systematically decreased the specific surface area and pore volume of the materials while promoting the formation of periodic domains, which are observed even at low organosilane molar percentages. Thermal characterisation via TGA/FT–IR/GC–MS revealed at least two main decomposition stages, with thermal stability following the order of 4–chlorophenyl > chloromethyl > 3–chloropropyl > 2–chloroethyl. This study focuses on kinetic and mechanistic insights in the thermal decomposition process through the Flynn–Wall–Ozawa isoconversional method and Criado master plots, using TGA/Differential Scanning Calorimetry (DSC) measurements under nitrogen at multiple heating rates (5, 10, 20, 30, and 40 K min^−1^), which revealed activation energies ranging from 53 to 290 kJ mol^−1^. Demonstrating that the chlorinated organosilane precursor directly controls both the textural properties and thermal behaviour of the resulting silica materials, with aromatic groups providing superior thermal stability compared to aliphatic chains. These quantitative kinetic insights provide a predictive framework for designing thermally stable hybrid materials while ensuring safe processing conditions to prevent hazardous volatile release.

## 1. Introduction

Hybrid organic–inorganic silica xerogels constitute a versatile class of advanced materials that combine the robustness and thermal stability of inorganic matrices with the chemical tunability of organic functional groups [1,2]. Among them, silica-based hybrid xerogels synthesised via the sol–gel method offer controllable porosity, low density, and adjustable surface chemistry [3,4], enabling applications in optical sensors, catalysts, membranes, and photonic devices [5,6,7]. Organofunctionalised xerogels containing chlorinated functional groups promote the formation of locally ordered domains through weak interactions (hydrogen bonding, dispersion forces, or π–π stacking) [8], thereby enhancing structural organisation and enabling fine control of microporosity [9,10,11].

Thermal stability is a critical property for these materials, as the degradation of organic moieties or cleavage of inorganic bonds may compromise structural integrity and functional performance, especially under prolonged thermal exposure [1,12,13]. The pyrolysis of organofunctionalised xerogels can also release hazardous volatile compounds, raising environmental and occupational health concerns [14,15]. Although previous studies have characterised the structure, morphology, porosity, and local order in chlorinated xerogel [9,10,11], quantitative kinetic analysis describing their composition mechanism remains scarce, especially for materials functionalised with organochlorinated groups.

Qualitative analysis of the thermal decomposition of silica xerogels containing a 10% molar content of organochlorinated groups (ClRTEOS, where ClR = ClM, ClE, ClP, or ClPh) has shown three principal mass loss stages and demonstrated that the decomposition pathways depend strongly on the nature of the substituents [16]. The release of several potentially hazardous volatile compounds during these processes further highlights the need for a quantitative kinetic and thermodynamic characterisation.

The broader literature shows two main approaches to the thermal evaluation of hybrid xerogels. One group of studies uses thermogravimetric analysis as a functional characterisation tool, extracting thermodynamic parameters (molar enthalpy change (Δ*H*) and the Gibbs energy (Δ*G*)) [17,18,19,20,21] or applying empirical kinetic models [22,23,24]. A second group performs formal kinetic assessments, but these are restricted to specific systems such as Eu^III^-doped xerogels [25], where isoconversional methods (Friedman or Flynn–Wall–Ozawa (FWO)) are applied. However, a comprehensive kinetic/thermodynamic framework for organochlorinated silica xerogels is still lacking, despite their technological interest and the confirmed release of hazardous volatiles during thermal decomposition.

This knowledge gap is particularly significant because defining safe operational temperature windows [26] requires understanding: (i) the apparent activation energies (*E*_α_); (ii) the reaction models, *f*(α), describing the degradation mechanisms; and (iii) the corresponding thermodynamic parameters (Δ*H* and Δ*G*). Therefore, determining this formation allows for reliable predictions of the long-term behaviour of the material [16].

The present study addresses this gap by providing a systematic kinetic and thermodynamic characterisation of the thermal decomposition of four organochlorinated silica xerogels (ClMTEOS, ClETEOS, ClPTEOS, and ClPhTEOS), using pure silica (TEOS) as a reference. The specific aims are as follows:

(i) Determine the *E*_α_ for each decomposition stage using the FWO isoconversional method [27,28], which does not require any prior assumption regarding the reaction mechanism.
(ii) Identify the most representative reaction model using the Criado master plot methodology [29], as recommended by the International Confederation of Thermal Analysis and Calorimetry (ICTAC) for solid-state reactions. According to the literature, the thermal degradation kinetics in inorganic materials typically conform to nucleation and growth models (An) [30]. In contrast, pyrolysis of organic matrices is best described by diffusion models (Dn) [31]. Meanwhile, n-order models (Fn) and geometrical contraction models (Rn) are applicable to all material types [32,33].
(iii) Calculate the relevant thermodynamic parameters (Δ*H* and Δ*G*) to provide insight into the endothermic nature of the decomposition processes.
(iv) Correlate the kinetic parameters with the previously identified [16] most abundant volatile species to establish safe operational temperature thresholds for each material. This kinetic data is crucial for assessing the thermal stability, predicting material performance in advanced applications (e.g., optical sensors and catalysts), and mitigating the potential emission of toxic compounds.

**Figure 1:**
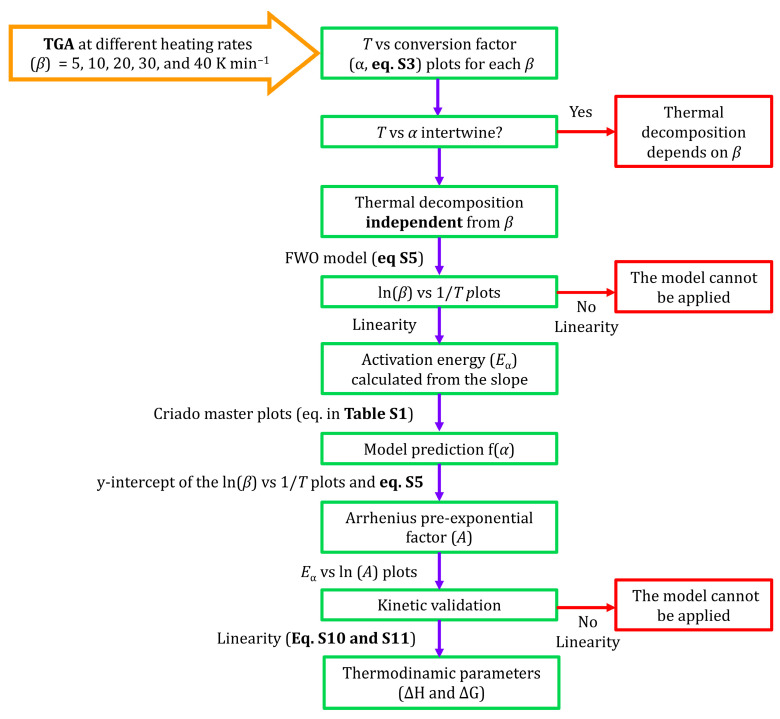
Scheme of the procedure followed for the kinetic analysis of the thermal decomposition of organochlorinated hybrid silica xerogels. Orange arrows represent the initial data, green boxes and purple arrows indicate the calculation route, and red arrows and boxes represent situations in which the kinetic model is not applicable.

Figure 1 schematises the methodological procedure followed in the present work, which consists of the following: (i) verifying that the *T* vs. conversion degree (*α*) plots for different heating rates (*β*) do not intertwine, (ii) applying the FWO method, (iii) calculating the *E*_α_, and (iv) determining the thermodynamic parameters (Δ*H* and Δ*G*).

By establishing a coherent kinetic–thermodynamic framework, this work provides essential insight into the thermal stability of chlorinated hybrid xerogels and supports their use in advanced technological applications.

## 2. Results and Discussion

### 2.1. Characterisation

The hybrid xerogel structure, degree of condensation, and texture were thoroughly characterised in previous works [9,10,11]. Table 1 compiles the relevant characterisation data that were pertinent to elucidating the behaviour observed in the kinetic analysis conducted in this study.

The incorporation of the organochlorinated species into the sol–gel synthesis directly influenced the textural and nanoscale structural characteristics of the resulting xerogels. Characterisation results revealed a correlation between the presence of ordered domains, the degree of condensation, and the material texture with the steric effects exerted by the organochlorinated moieties of the triethoxysilane precursors during the condensation step of the sol–gel synthesis [34].

On one hand, when a molar percentage of 10 mol% of organochlorinated precursor was employed, the bulky chloropropyl moiety induced the formation of polyhedral silsesquioxanes (POSSs) within the amorphous silica matrix of ClPTEOS, as demonstrated by the presence of a diffraction maximum at 5.8°, whereas the chloromethyl moiety did not do so in ClMTEOS. This greater order translates into a lower specific surface area (*a*_BET_) and reduced pore volumes for ClPTEOS compared to ClMTEOS, due to POSS being composed of the most condensed Si species (Q^3^, Q^4^, and T^3^), which restricts porosity development during crosslinking.

On the other hand, although the chloroethyl moiety is less voluminous than chloropropyl, the PXRD diffraction pattern of ClETEOS indicated that chloroethyl moieties promoted the formation of the highest proportion of POSS among the xerogels studied. This is attributed to the rigidity of the ethyl chains, whose limited degrees of freedom compared to chloropropyl exponentially increase the time required for gelation, thus favouring the formation of the kinetically unfavourable POSSs. As a result, ClETEOS is a highly condensed material, in which the abundance of T^3^ species matches that of Q^4^ species, and does not adsorb N_2_ due to the collapse of its porosity.

Finally, ClPhTEOS, the xerogel synthesized using a chlorinated aryl moiety, presented a diffraction maximum at 3.6°, although its intensity was lower than that of ClETEOS and ClPTEOS. This indicates that the chlorophenyl moiety has a lower capability of inducing order than chloropropyl and chloroethyl moieties, likely due to its planar geometry and its capacity to establish π–π interactions that avoid complete incorporation into the silica matrix. This is consistent with the degree of condensation of this material, since T^3^ species are less abundant than T^2^ species, with its laminar morphology observed by FE–SEM microscopy, and with its higher *a*_BET_ and pore volumes compared to ClPTEOS and ClETEOS.

In summary, the organochlorinated moieties of the precursors can be ranked according to their ability to induce local order in the silica matrix, decrease *a*_BET_ and pore volume, and generate more highly condensed materials, following the order of 2–chloroethyl > 3–chloropropyl > 4–chlorophenyl > chloromethyl.

### 2.2. Thermal Analysis

**Figure 2:**
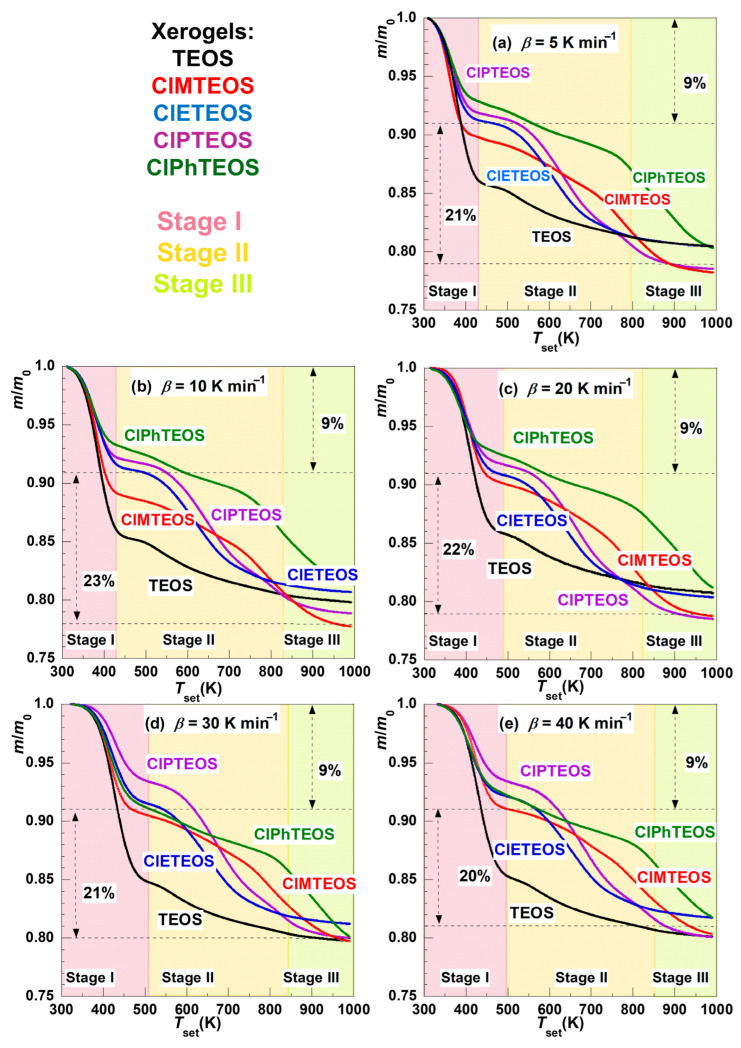
Evolution of the normalised mass as a function of the programmed temperature for the TEOS reference xerogel (black line) and the four organochlorinated ClRTEOS materials (lines: ClMTEOS, red; ClETEOS, blue; ClPTEOS, purple; and ClPhTEOS, green) at a heating rate of (**a**) 5 K min^−1^, (**b**) 10 K min^−1^, (**c**) 20 K min^−1^, (**d**) 30 K min^−1^, and (**e**) 40 K min^−1^. The different decomposition stages are delimited with different colours: Stage I: lilac, Stage II: peach, and Stage III: green lime.

Figure 2 depicts the thermal evolution of the normalised mass loss (*m*_loss_) of the reference xerogel (synthesised using only TEOS) and the four organochlorinated hybrid xerogels ClMTEOS, ClETEOS, ClPTEOS, and ClPhTEOS (with 10% molar content of the corresponding ClRTEOS precursor) for a heating rate (*β*) of 5, 10, 20, 30, and 40 K min^−1^. The corresponding *m*_loss_ data for each decomposition stage are listed in Table S1. The different organic moieties of the ClRTEOS materials produced different behaviours during their decomposition at constant heating rates. Nevertheless, at least two distinct thermal decomposition stages are evident in all cases.

While the total *m*_loss_ during the overall process was approximately 20–22% in all cases, the relative mass fractions of the individual decomposition stages differed significantly between TEOS and ClRTEOS. Notably, the overall stability of the reference material, TEOS, is enhanced by the incorporation of just 10% of organochlorinated derivatives, being noticeable for the chloropropyl and chlorophenyl moieties. This enhancement can be attributed to a modified porous texture in the resulting xerogels, whereby the inclusion of such precursors reduces both the pore size distribution and the specific surface area, cancelling the mesoporous domain present in TEOS and diminishing the pore volume [16].

For the reference material, the first stage (desolvation) constitutes the primary decomposition process (Table S1) as this *m*_loss_ is associated with the release of physiadsorbed solvent (water and ethanol) from the surface, as characterised in a previous work [16]. This behaviour is attributed to the fact that the TEOS exhibits a pore size distribution with a higher amount of pore volume centred in the mesoporous region and has also higher hydrophilic character, due to the greater number of surface Si–OH groups, in contrast to the organochlorinated xerogels, which contain hydrophobic chlorinated alkyl or aryl moieties and their vast majority of their pore volume is centred in the microporous region and higher condensation degree with more abundant siloxane bonds. The principal *m*_loss_ for the ClRTEOS materials occurs at higher temperatures (from 450–500 K up to 900–1000 K) and is associated with the decomposition of organic fractions. The decomposition profiles of the hybrid xerogels prepared using 3–chloropropyl and 2–chloroethyl-containing triethoxysilane precursors (ClPTEOS and ClETEOS, respectively) are similar and display a steep *m*_loss_ extending up to ca. 750 K as the second stage. These profiles differ from those of the materials prepared with the chloromethyl and 4–chlorophenyl derivatives (ClMTEOS and ClPhTEOS, respectively), which are alike and exhibit a progressive, extensive *m*_loss_ that reaches higher temperatures between ca. 750 and 800 K instead.

In our previous investigation on the products released during the pyrolysis of organochlorinated xerogels [29], we could identify small molecules like CO_2_ or HCl (accompanied by chloroethane) for ClMTEOS and ClETEOS, respectively, and larger species for ClPTEOS (cyclopropane, chloroethane) and ClPhTEOS (propene, chlorobenzene, and a collection of chlorinated aromatics). The nature of the released vapours suggests that it is governed by dechlorination and chain–decomposition reaction mechanisms.

For all hybrid xerogels except ClETEOS, the thermal decomposition proceeds up to temperatures above 900 K with a further *m*_loss_. The vapours released were identified to be mainly chloromethane for ClMTEOS, cyclopropane for ClPTEOS, and chlorobenzene for ClPhTEOS [16]. In all cases, these molecules were accompanied by benzene and other heavier molecules, such as alkenes and dienes (hexadiene for ClMTEOS, butene and cyclopentadiene for ClPTEOS) or aromatic compounds (naphthalene for ClMTEOS, toluene for ClPTEOS, and styrene for ClPhTEOS). These findings align with the fact that the formation of heavy organic compounds through chain cyclisation and aromatisation requires higher decomposition temperatures. Thus, the decomposition behaviour of the hybrid xerogels differs markedly compared to the TEOS reference, which constitutes 90% of the composition in the ClRTEOS materials, indicating that the silica matrix is more thermally stable, whereas the chlorinated organosilane moieties are the most thermally susceptible.

The decomposition of the ClRTEOS materials at different heating rates faster than 5 K min^−1^ (*β* = 10, 20, 30, and 40 K min^−1^) exhibits analogous decomposition profiles with the upper limits of the decomposition stages shifting toward higher temperatures as *β* increases (Figure 2b–e). The *m*_loss_ in the initial desolvation stage decreases slightly with increasing heating rate, except for TEOS and ClPhTEOS. Both materials undergo an increase in *m*_loss_ within this stage, which is remarkable in the case of TEOS. Increasing *β* causes thermal decomposition products to be released rapidly and the energy required for decomposition to be reached more quickly, as observed for these two xerogels (TEOS and ClPhTEOS). In the second stage, the *m*_loss_ decreases slightly as the heating rate *β* becomes faster in all materials except TEOS. In contrast, slight increases of *m*_loss_ with *β* are observed within the third stage, ClPhTEOS having the biggest *m*_loss_.

**Figure 3:**
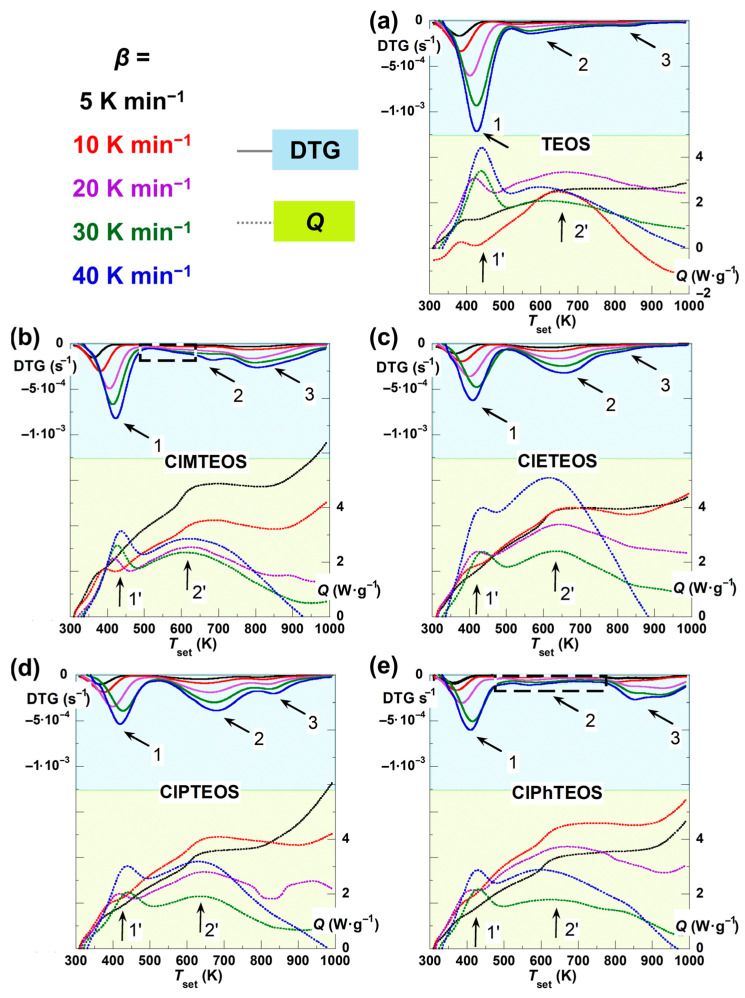
Evolution of the first-time derivative of the thermogravimetric curves (DTG, solid top curves and blue shading) and the heat flow (*Q*, bottom dotted curves and green lime shading) as a function of the programmed temperature for (**a**) the TEOS reference, and the hybrid xerogels: (**b**) ClMTEOS, (**c**) ClETEOS, (**d**) ClPTEOS, and (**e**) ClPhTEOS.

Figure 3 shows the thermal evolution of both the first time-derivative of thermogravimetric curves (DTG, top solid curves with blue shading) and the normalised heat flux (*Q*, bottom dotted curves with green lime shading) of the TEOS reference and the four hybrid xerogels recorded at five different values of *β*. The DTG curves exhibit up to three minima, each one corresponding to one of the three decomposition stages. In the case of TEOS, the *m*_loss_ above 750 K is negligible, thus the third stage cannot be confirmed from the *Q* data. For ClPhTEOS and ClMTEOS, the second stage displays a notably flat profile (enclosed in a dashed box in Figure 3), which may compromise the accuracy of subsequent kinetic calculations.

In the *Q* curves (green line area in Figure 3), the different endothermic signals are indicated on the graphs using arrows. For all the xerogels studied, the first two maxima are well defined and clearly distinguishable, while the third signal, in most cases, is not as clear because it is shadowed by that of the second stage and becomes difficult to distinguish with enough definition. This third signal is best observed in the case of ClPTEOS, with a maximum of ca. 900 K. In all instances, the maxima corresponding to the second thermal stage display considerable width, suggesting that they likely encompass multiple overlapping processes of different natures.

### 2.3. Kinetic Analysis

**Figure 4:**
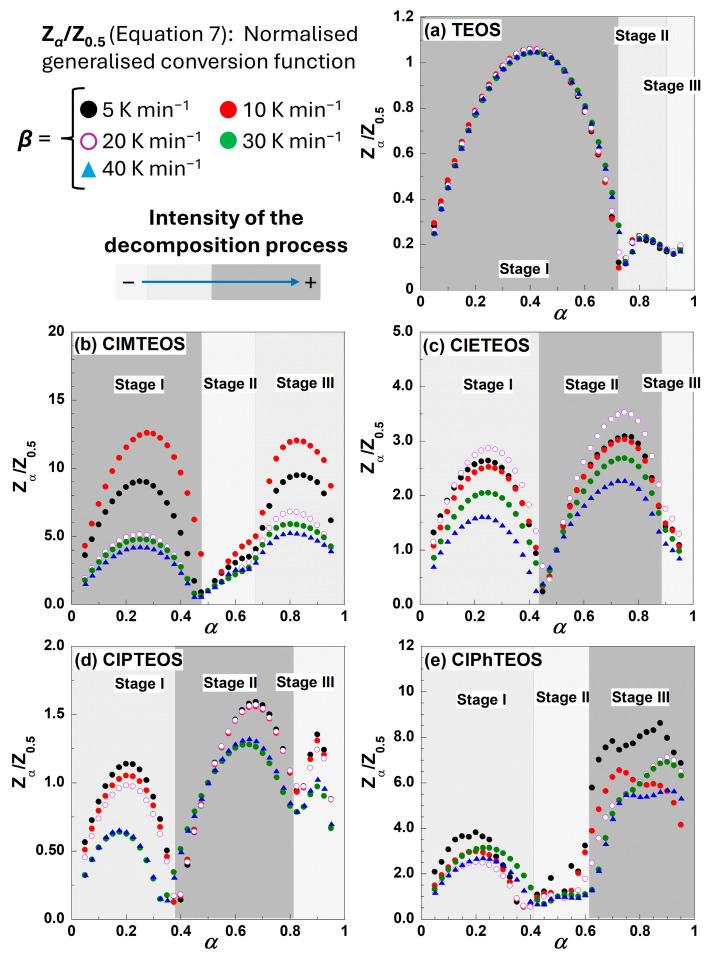
Criado master plots showing the evolution of the normalised generalised conversion function (Z*_α_*/Z_0.5_) with the conversion degree *α* for the (**a**) TEOS reference and the hybrid (**b**) ClMTEOS, (**c**) ClETEOS, (**d**) ClPTEOS, and (**e**) ClPhTEOS xerogels.

To perform a preliminary evaluation of the mechanism governing the global decomposition process, normalised Criado master plots were constructed for all xerogels after confirming that their *T* vs. *α* plots for the five different heating rates do not intertwine significantly. The Criado master plots were obtained by following the methodology described in Section 4.1 (see from Equation (1) to (7)), and the results are shown in Figure 4.

The simplest behaviour is found in the case of TEOS, with the first stage serving as the principal governing process. For the organochlorinated xerogels, the first stage occurs at a conversion degree of approximately α = 0.4 (40% of the total *m*_loss_), whereas the second and subsequent stages differ markedly in both the shape and extent of decomposition. The transition between the second and the third stages is not well defined, especially for ClMTEOS and ClPhTEOS, as the Z*_α_*/Z_0.5_ curve does not reach a value of zero, indicating the initiation of an alternative mechanism. This transition is almost imperceptible for TEOS and ClETEOS.

**Figure 5:**
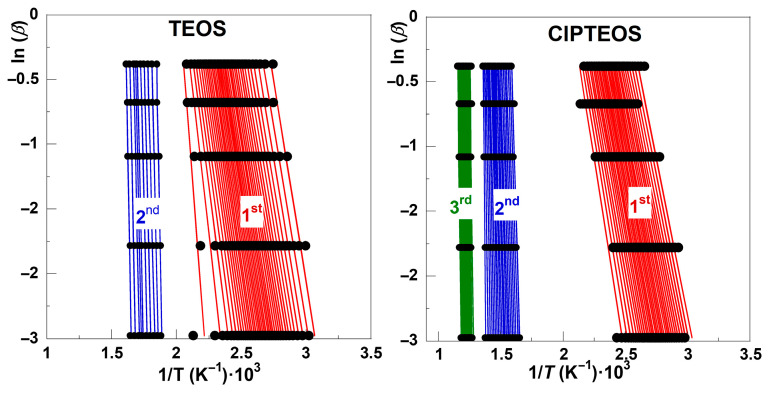
Linearity of the FWO method for the thermal decomposition of TEOS and ClPTEOS using ln(*β*) vs. 1/*T* plots within the *α* = 0.05–0.95 range with increments of 0.025. Red, blue, and green symbols correspond to the first, second, and third decomposition stages, respectively.

To determine the kinetic decomposition mechanism using an isoconversional method, the first task was to identify the temperature intervals over which each stage develops and to define the fraction of mass lost in each case. For this work, this identification was guided by the shaded regions marked in Figure 4, determined by applying Equation (4). According to the FWO method and by plotting ln(*β*) against 1/*T* within the *α* = 0.05–0.95 range using increments of 0.025 [35], linear trends with regression coefficients higher than 0.97 were obtained for TEOS and ClPTEOS, as shown in Figure 5 (Figure S3 collects the ln(*β*) vs. 1/*T* plots for the remaining three xerogel samples). The corresponding slopes are parallel, except within those temperature intervals where different mechanisms overlap, denoting the independence of the decomposition with *β*. This finding indicates a good agreement for the best fit to calculate the kinetic parameters.

Although *α* varies from 0 to 1 in both graphs in Figure 5, the sensitivity of the study may not be equivalent within the whole range. The sensitivity will be linked to the relative *m*_loss_ of each stage, which is quite different for each xerogel sample studied in this work, according to the results gathered in Figure 2. For example, the *m*_loss_ associated with the first and second decomposition stages of TEOS corresponds to 14% and 6%*,* respectively, whereas *m*_loss_ values of 8% and 12% are observed in the case of ClPTEOS, the third stage encompassing an additional *m*_loss_ of 1%. The slope of the ln(*β*) vs. 1/*T* lines increases as the stages evolve, and this trend was also found for the rest of the organochlorinated xerogels (Figure S3).

**Figure 6:**
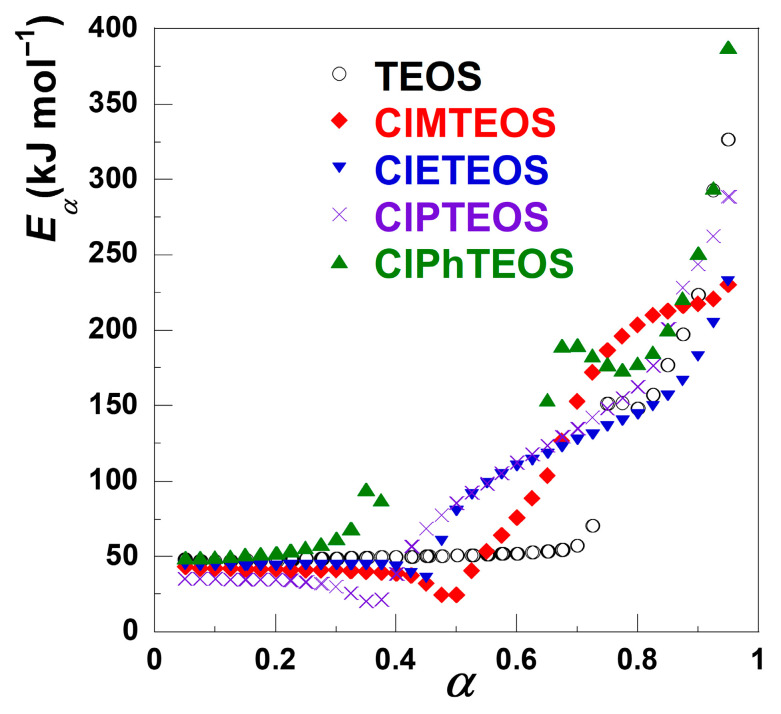
Variation of the activation energy with α obtained from the FWO method for the TEOS reference and the four ClRTEOS materials.

Figure 6 shows the variation of the activation energy (*E*_α_) with α obtained from the FWO method, where *E*_α_ exhibits different values for each decomposition stage, confirming the multi-step nature of the thermal decomposition [36]. The progressive increase in *E*_α_ with each subsequent stage indicates stable decomposition reactions and consistent disorder degree [37].

Table 2 summaries the minimum and the maximum values of *E*_α_ for each decomposition stage, which were calculated from the slope of the FWO plots using the methodologies described in Section 4.1. The obtained values agree with those found in the literature, considering that the errors are similar (in the 5–10% order), mainly due to the fitting of d*α*/d*t* [38].

For the first stage, the *E*_α_ value ranges from 35 to 60 kJ mol^−1^, which relates to the release of the surface physiadsorbed solvent species. This process shows a direct correlation with the material textural properties, exhibiting higher *E*_α_ values for samples with greater mesoporous distribution [9,10,11]. The second stage covers the thermal decomposition of the chlorinated organosilane moieties and further condensation of the Si–OH groups. For this stage, the *E*_α_ values increase progressively with the thermal decomposition evolution, likely due to reduced accessibility of free silanol groups within the matrix. For the organochlorinated xerogels, the dehydroxylation reaction becomes less significant due to the lower amount of TEOS used in their preparation, resulting in silica matrices with a lower abundance of Si–OH groups. However, the use of a 10% molar content of ClRTEOS precursors introduces additional, alternative decomposition pathways due to the organochlorinated moieties. Furthermore, the collapse of the matrix porosity reduces the diffusion rate of decomposition products [22]. Notably, the determination of *E*_α_ was precluded for ClMTEOS and ClPhTEOS due to the markedly flat DTG evolution in their second decomposition stages (marked with dashed boxes in Figure 3). Kappert et al. reported that the *E*_α_ values for the dehydroxylation reaction are highly dependent on the conversion degree, ca. 150–300 kJ mol^−1^, whereas the degradation of the functional group in organosilica matrices requires slightly lower values, 160–190 kJ mol^−1^ [22].

Crucially, both the second and third decomposition stages (occurring above 420–500 K, depending on *β*) generate the most hazardous vapour species released from the thermal decomposition. Table 3 summarises the predominant species identified during the thermal decomposition of the ClRTEOS xerogels in recent work [29], along with the characteristic temperature ranges for each mass loss stage. The first stages are associated with the diffusion process of ethanol out of the silica network. The second stages originate mainly from the dehydroxylation of the material, together with minor decomposition of the organic fragments from ClRTEOS moieties. The Si–C bonds break at the same time, bringing out the isolation of additional siloxane (Si–O–Si) bonds, which hinders the convergence of silanol groups and increases the dehydroxilation activation energy as a result. Hence, *E*_α_ increases with the conversion degree, and a higher value of energy is required for the thermal decomposition to proceed, in agreement with the siloxane bonds established between the silanols. The third stage is only observed for ClMTEOS, ClPTEOS, and ClPhTEOS and involves the highest *E*_α_ values, most likely because it relates to cyclisation and aromatisation of the organic fragments. Based on these results, ClPTEOS and TEOS demonstrated the highest thermal stability, while ClPhTEOS showed the lowest stability among the studied materials.

The values of Δ*H* calculated from Equation (8) (Figure S4) were positive for all stages, reflecting the well-known, endothermic nature of the decomposition process. The numerical values were slightly lower (ca. 3–8 kJ mol^−1^) than those of the corresponding *E*_α_. The values of Δ*G* were also calculated from Equation (9) (Figure S5), resulting in positive high values as corresponds to non-spontaneous processes, which increase with the temperature required for the thermal decomposition.

**Figure 7:**
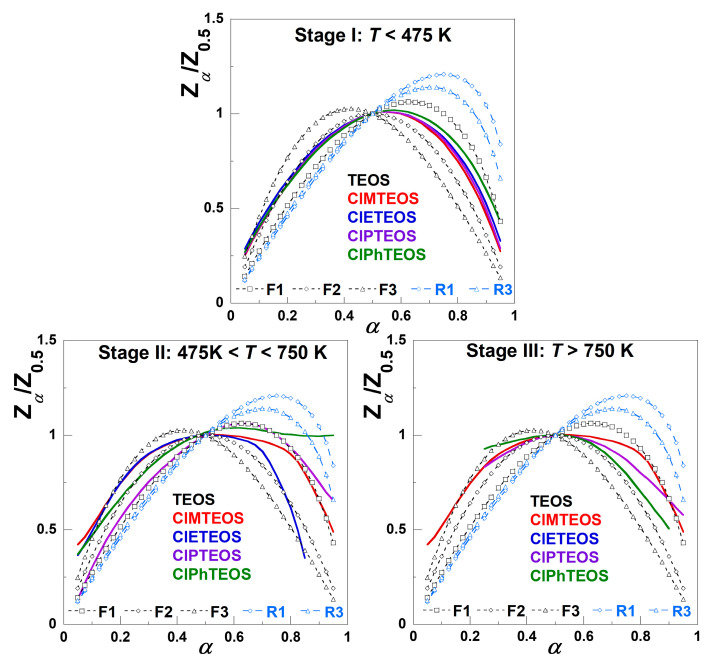
Criado master plots with the data from the isoconversional method (F1, F2, F3, R1, and R3 models in Table S2) for the TEOS reference and the hybrid xerogels ClMTEOS, ClETEOS, ClPTEOS, and ClPhTEOS.

The Criado master plot technique was employed to evaluate the most probable reaction model for all samples. Equation (7) was used to plot the theoretical and reduced rate curves of Z_α_/Z_0.5_ against α for each decomposition stage of each sample using the mathematical expressions of the main kinetic models collected in Table S2. The best-fitting model was that of nth order kinetics, Fn. Figure 7 shows the experimental Criado master plots at *β* = 5 K min^−1^ for the TEOS reference and the four ClRTEOS materials compared to the predicted values for first, second, and third order, *f*(*α*) = (1−α) n, and for contracting geometrical models, being n = 1/2 for contracting area and n = 2/3 for contracting volume.

The comparison between the experimental and predicted Criado master plots for other considered models for solid-state reactions is included in Figure S6. All the hybrid xerogels display a similar experimental behaviour, and the Fn order models afforded the best fits. For the first stage, the experimental curve overlaps the F2 model when *α* is lower than 0.5, whereas at higher *α* values, the experimental evolution lies between the first and second orders. The behaviour for the second stage depends on the specific nature of the chlorinated organosilane moiety. While ClPTEOS follows the F1 model for almost the whole *α* range, the best fitting at *α* values lower than 0.5 corresponds to F2 for ClPhTEOS, the xerogels with the shorter alkyl chains (ClMTEOS and ClETEOS) deviating from the F3 model toward the F2 at low *α* values just above ca. 0.20. The trends above *α* = 0.5 are complex and do not adjust to any model except for ClMTEOS, which approaches the F1 model at high conversion degree. In the third stage, the analysis is more complex, and the evolution for all the xerogels is included between the models F1 and F2, which could be due to the scarce *m*_loss_ involved and the complex decomposition pathways resulting in silicon oxycarbide ceramics.

The selection of the best-fit reaction model was based on the calculation of the non-regression coefficient (R^2^) and the lowest value of the root mean square error (RMSE). In the first stage, the evolution is common for all matrices, with the second order being the one that fits the best. In the second and third stages, the thermal degradation is more complex, as illustrated by the overlapping mechanisms in Figure 7. A reaction order greater than one is the result of the decrease in the decomposition rate with increasing *m*_loss_. In heterogeneous reactions such as those in the present study, the reaction could be diffusion-controlled across an unstable interface that is reducing by a sintering process, or the accumulation of products at the interface could even lead to an increase in the diffusion resistance along the decomposition process.

## 3. Conclusions

This study establishes quantitative structure-property relationships governing the thermal stability of organochlorine-functionalised silica xerogels, revealing a fundamental and counterintuitive principle: thermal resistance inversely correlates with the capability of the organic moieties to induce the formation of ordered domains. The validated kinetic models and identification of specific volatile decomposition products provide essential tools for predicting thermal performance under processing conditions and assessing potential environmental hazards. Beyond the specific materials examined, this work introduces a predictive framework for rational design of hybrid organic-inorganic materials where thermal stability requirements must be balanced against structural functionality. The findings enable the selection of materials with the appropriate organic functionalities based on target application temperatures and to optimize synthesis conditions for desired thermal properties, thereby accelerating the development of advanced xerogel materials for high-temperature applications in catalysis, separations, and protective coatings.

## 4. Materials and Methods

### 4.1. Materials

Tetraethoxysilane (purity > 99%), (chloromethyl)triethoxysilane (purity > 95%), (3–chloropropyl)triethoxysilane (purity > 95%), and (4–chlorophenyl)triethoxysilane (purity > 97%) were supplied by Sigma-Aldrich (St. Louis, MO, USA) while (2–chloroethyl)triethoxsysilane (purity > 95%) was obtained from Fluorochem Ltd. (Glossop, UK). Absolute ethanol (Emsure®, Merck, Darmstadt, Germany) and hydrochloric acid (HCl, 37% *w*/*w*, Sigma-Adrich) were purchased. All chemicals were used without further purification.

### 4.2. Synthesis of the Organochlorinated Xerogels

**Figure 8:**
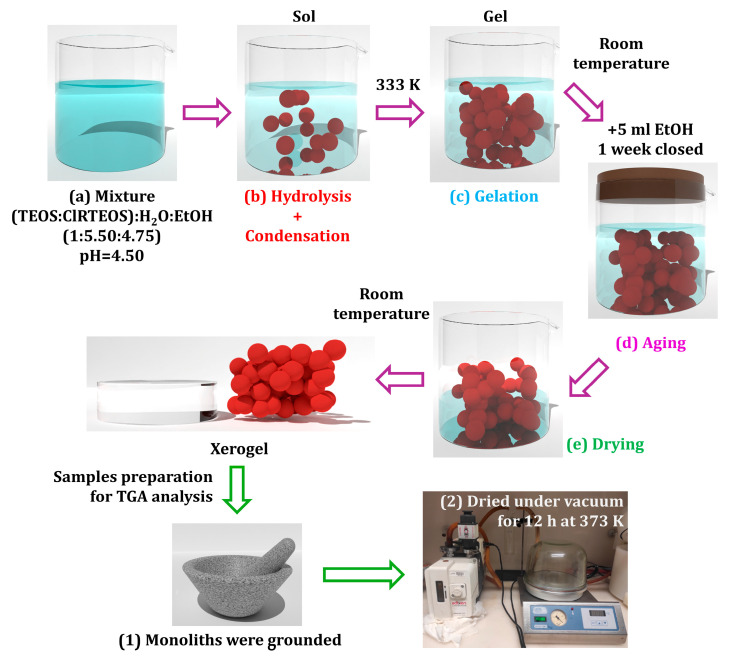
Scheme of the synthesis and sample preparations of the reference material and the four organochlorinated xerogels.

Monoliths of the pure silica reference material (TEOS) and four organochlorinated hybrid silica xerogels (ClMTEOS, ClETEOS, ClPTEOS, and ClPhTEOS) were synthesised as described in previous works [14,15,16] and illustrated in the diagram of Figure 8.

The xerogels were prepared through the sol–gel method in acidic conditions (pH = 4.5, adjusted by dropwise addition of HCl 0.05 M) using blends of tetraethoxysilane (TEOS) and the corresponding ClRTEOS triethoxysilane (ClR substituent = ClM, chloromethyl; ClE, 2–chloroethyl; ClP, 3–chloropropyl; ClPh, 4–chlorophenyl) with a 90:10 molar ratio, where the mixture of precursors is added dropwise to a solution of ethanol and miliQ water following the 0.90:0.10:5.50:4.75. (TEOS:ClRTEOS:EtOH:H_2_O) molar ratio. Only 10% molar percentage of ClRTEOS was employed since, in previous works, it was determined that this content is enough for the study of the effect of the incorporation of such moieties into silica materials without compromising their structural integrity. Once the sols were obtained, the closed vessels were introduced into a thermostatized oven (J.P Selecta S.A., Barcelona, Spain) at 333 K until gelation (when sols do not move when the container is tilted). Afterwards, the lid was opened, and 5 mL of ethanol was added to strengthen cross-linking and cure the gel at room temperature (~298 K) for one week. Finally, the lid was removed, and the gels were dried under atmospheric conditions.

The monoliths were ground and dried under vacuum for at least 12 h to extract their surface moisture before performing the thermogravimetric experiments (TGA/DTG), which were carried out under a N_2_ flow of 40 mL min^−1^ using a Mettler Toledo TGA/DSC 3+ series thermogravimetric analyses (Mettler Toledo, Greifensee, Switzerland). Samples of approximately 15 mg were placed in 70 μL sapphire crucibles for thermal analysis from 303 to 1000 K at constant heating rates of 5, 10, 20, 30, and 40 K min^−1^.

### 4.3. Methodology of Kinetic Studies

For a single-step process, the reaction rate can be represented by Equation (1):

(1) dαdt=kT·fα=Aα·e−EαR·T·fα

where *k*(*T*) is the kinetic constant as a function of absolute temperature *T*; *f*(*α*) denotes the kinetic model function that depends on the reaction mechanism and the conversion degree (*α*); *A*_α_ is the Arrhenius pre-exponential factor; *E*_α_ represents the apparent activation energy. The kinetic model is defined in terms of the global conversion *α*, which represents the mass fraction volatilised. For each decomposition stage, *α* can be defined as follows:

(2) $$\alpha_{i}=\frac{m_{o,i}-m_{T}}{m_{o,i}-m_{f,i}}$$

where *m*_o,i_, *m_T_*_,i_, and *m*_f,i_ designate the initial, instantaneous, and final masses within each stage, respectively. Equation (3) displays the integral form of the kinetic model function, *g*(*α*):

(3) gα=∫0αdαfα=Aα·∫0αe−EαR·T·dt 

This integral does not have an exact analytical solution, but it can be solved by numerical approximation methods or by using other approximations proposed in the literature. For example, the FWO model [27,28] that employs Doyle’s approximation [39] can be applied for 20 ≤ *E_α_*/*R T* ≤ 60 when the thermal decomposition is carried out at a constant value of the heating rate *β*. Under these conditions, Equation (3) can be rearranged as follows:

(4) $$ln\beta =\ln\left(\frac{A_{\alpha }\cdot E_{\alpha }}{R\cdot g(\alpha )}\right)-5.331-1.052\cdot \frac{E_{\alpha }}{R}\cdot \frac{1}{T_{\alpha }}\;$$

For isoconversional data, g(*α*), *A_α_*, and *E*_α_ have constant values. Thus, by registering the temperatures necessary (*T_α_*) to reach a particular decomposition degree *α* at different heating rates *β*, the activation energy *E_α_* can be calculated from the slope of linear ln *β* vs. 1/*T*_*α*_ plots (*m* = 1.052·*E*_*α*_/*R*). The application of the isoconversional treatment allows for obtaining *E_α_* without considering any reaction model *f*(*α*).

For thermal processes developed at a constant heating rate (*β* = d*T*/d*t*), Equation (3) can be written as the following temperature integral, having an analytical solution:

(5) gα=∫0αdαfα=Aαβ·∫0αe−EαR·T·dT=Aα·R·Tα2Eα·β·exp−EαR·Tα

In this context, Criado proposed the use of the variable *Z*(*α*), which is defined as the product of differential and integral model contributions, *f*(*α*)*·g*(*α*), and the values of which can be easily calculated for each *α* from experimental TGA/DTG data [24]. The expression of *Z*(*α*) is given in Equation (6):

(6) $$Z\left(\alpha \right)=f\left(\alpha \right)\cdot g\left(\alpha \right)=\frac{R\cdot {T_{\alpha }}^{2}}{E_{\alpha }\cdot \beta }\cdot \frac{d\alpha }{dt}$$

The mathematical expressions of *f*(*α*) and *g*(*α*) are well known for numerous kinetic models, and Table S2 collects the equations for the main ones. Applying these expressions allows the calculation of the theoretical values of Z*_α_* for each *α*. A Criado master plot consists of a representation against *α* of the values of the variable *Z*(*α*), either experimental or theoretical, normalized, with the corresponding value at *α* = 0.5 according to Equation (7):

(7) ZαZα=0.5exp.=TαTα=0.52dαdtαdαdtα=0.5

The comparison of experimental and theoretical data by means of Criado master plots allows identifying the predominant kinetic mechanism within a thermal decomposition process, of organochlorinated hybrid silica xerogels in this case.

The implementation of the FWO method has important limitations, the first and main one being that the thermal decomposition cannot depend on *β* Other limitations relate to the linear behaviour of the ln *β* vs. 1/*T* or ln *β* vs. ln *A* plots. Figure 1 schematises the calculation procedure followed in this work for implementing and validating the FWO method. Once the validation is satisfactorily completed, the molar enthalpy (Δ*H*) and Gibbs energy (Δ*G*) changes can be calculated through Equations (8) and (9) [40,41]:

(8) $$\Delta H=E_{\alpha }-R\cdot T_{m}$$

(9) $$\Delta G=E_{\alpha }+R\cdot T_{m}\cdot ln\left(\frac{K_{B}\cdot T_{m}}{h\cdot A}\right)$$

where *T*_m_ is the maximum decomposition temperature, *K*_B_ is the Boltzmann constant (1.381·10^−23^ J K^−1^), and *h* is the Planck constant (6.626·10^−34^ J s).

## Figures and Tables

**Table 1:** Characterisation result of reference material, TEOS, and the hybrid xerogels synthesised with 90:10 molar percentage of tetraethoxysilane: organochlorinated by PXRD, ^29^Si NMR studies, and the adsorption-desorption isotherm with N_2_ at 77 K. The details of the characterisation methods are described in previous works [9,11].

Material	Gelation Time	Structure (PXRD)	Degree of Condensation (^29^Si NMR)		Textural Parameters (N_2_ Adsorption-Desorption Isotherm at 77 K)				Ref.
					*a*_BET_	*V*_micro_	*V*_meso_	*V*_total_	
	h	2*θ* (°)	Intensity (a.u.)	T^i^/Q^i^ Relative Abundances	(m^2^ g^−1^)	(cm^3^ g^−1^)			
TEOS	5	a	a	Q^3^ > Q^4^ > Q^2^	697	0.33	0.07	0.41	[9,11]
ClMTEOS	8	a	a	Q^3^ > Q^4^ > T^3^ ≈ Q^2^ > T^2^	662	0.30	0.02	0.32	[9,10]
ClETEOS	257	6.76	16,568	Q^3^ > T^3^ ≈ Q^4^ ≈ Q^2^ ≈ T^2^	b	b	c	b	[9,10]
ClPTEOS	41	5.80	5514	Q^3^ > Q^4^ > T^3^ ≈ T^2^ ≈ Q^2^	132	0.05	c	0.05	[9,10]
ClPhTEOS	23	3.60	2112	Q^3^ > Q^4^ > Q^3^ > T^2^ > T^3^	367	0.15	c	0.15	[11]

^a^ The diffraction maximum was not observed; ^29^Si NMR: ^29^Si nuclear magnetic resonance; Q and T are the Si atoms belonging to tetraethoxysilane and organochlorinated precursors, respectively, and *i* is the number of Si–O–Si bridges in each silicon atom; *a*_BET_: Specific surface area calculated by BET method; *V*_micro_: Micropore volume obtained from Dubinin–Raduskevich; *V*_meso_: Calculated from isotherm (*V*_meso_ = *V*_total_ − *V*_macro_ − *V*_micro_); *V*_total_: Total pore volume obtained from isotherm at *p*/*p*° = 0.95; ^b^ Samples did not adsorb; ^c^ Could not be calculated.

**Table 2:** Minimum and maximum values of the activation energy (*E_α_*) for each stage in the thermal decomposition of the TEOS reference and the four hybrid ClRTEOS materials.

Xerogel	*E*_α_ (kJ mol^−1^)		
	Stage I	Stage II	Stage III
TEOS	53–60	92–240	–
ClMTEOS	35–43	–	147–177
ClETEOS	47–53	93–158	–
ClPTEOS	41–42	74–186	205–252
ClPhTEOS	53–54	–	233–289

**Table 3:** Assignment of thermal decomposition and the most abundant species in the studied xerogels.

Xerogel	Stg.	Predominant Species * [16]	Temperature Intervals (*β* = 5 K min^−1^)	Process
TEOS	I	Ethanol	*T* < 480 K	Desolvation
	II	Ethanol	*T* = 480 K–780 K	Dehydroxylation/Ethoxy group
ClMTEOS	I	Ethanol	*T* < 470 K	Desolvation
	II	[a]	*T* = 470 K–740 K	Dehydroxylation/Ethoxy group
	III	**Chloromethane**/Naphthalene	*T* > 740	Dechlorination/Aromatisation
ClETEOS	I	Ethanol	*T* < 470 K	Desolvation
	II	**Hydrochloric acid**/Chloroethane	*T* = 470 K–730 K	Dehydroxylation/Ethoxy group/Dechlorination
ClPTEOS	I	Ethanol	*T* < 485 K	Desolvation
	II	**Cyclopropane**/Chloroethane	*T* = 485 K–760 K	Dehydroxylation/Ethoxy group/Dechlorination
	III	**Cyclopropane**/Butene/Toluene	*T* > 760 K	Dechlorination/Aromatisation
ClPhTEOS	I	Ethanol	*T* < 420 K	Desolvation
	II	**Chlorobenzene**	*T* = 420 K–780 K	Dehydroxylation/Ethoxy group/Dechlorination
	III	**Chlorobenzene**/Styrene	*T* > 780 K	Dechlorination/Aromatisation

* The most abundant species identified in the GC–MS are highlighted in bold. [a] The released species could not be unequivocally identified.

## Data Availability

The data presented in this study are available on request from the corresponding author.

## Supplementary Materials

The following supporting information can be downloaded at: https://www.mdpi.com/article/10.3390/gels12010002/s1, Figure S1: FT–IR obtained from the reference and the four organochlorinated xerogels at the point of maximum thermal decomposition identified in the GC–MS analysis [29]; Figure S2: Thermal evolution of the relative abundance of the organic fragments detected in the vapours from the pyrolysis of the reference and the organochlorinated xerogels (a–e) at different decomposition stages and the most relevant species identified [29]; Figure S3: Linearity of the FWO method for the thermal decomposition of ClMTEOS, ClETEOS and ClPhTEOS using ln(*β*) vs. 1/*T* plots within the *α* = 0.05–0.95 range with increments of 0.025; Figure S4: Dependence of the molar enthalpy change with α for the TEOS reference and the four organochlorinated ClRTEOS xerogels; Figure S5: Dependence of the Gibbs energy change with α for the TEOS reference and the four organochlorinated ClRTEOS xerogels; Figure S6: Criado master plots for the three decomposition stages of the TEOS reference and the four organochlorinated ClRTEOS materials using the different Pn, An, Dn, and Rn models collected in Table S2; Table S1: Values of the mass loss for each heating rate at each interval of temperature for studied materials; Table S2: Fitting performance of various kinetic models with different values of *f*(*α*) and *g*(*α*) [16].

## Abbreviations

The following abbreviations are used in this manuscript:

TGA	Thermogravimetric analysis
FT–IR	Infrared spectroscopy
GC–MS	Gas chromatography-mass spectrometry
ClRTEOS	Chlorinated xerogels
TEOS	Tetraethoxysilane
ClMTEOS	(Chloromethyl)triethoxysilane
ClETEOS	(2-Chloroethyl)triethoxysilane
ClPTEOS	(3-Chloropropyl)triethoxysilane
ClPhTEOS	(2-Chlorophenyl)triethoxysilane
SEM	Scanning Electron Microscope
^29^Si NMR	^29^Si Nuclear Magnetic Resonance
Δ*H*	Variation of the molar enthalpy
Δ*G*	Gibbs energy
FWO	Flynn–Wall–Ozawa
Fn	n-Order models
ICTAC	International Confederation of Thermal Analysis and Calorimetry
*m*_loss_	Mass loss
*α*	Conversion factor
An	Nucleation and growth models
Dn	Diffusion models
Rn	Geometrical contraction
*β*	Heating rate
PXRD	Powder X–ray Diffraction
*a*_BET_	Specific surface area calculated by Brunauer–Emmet–Teller method
V_meso_	Volume of mesoporous
V_micro_	Volume of microporous
V_total_	Total volume
*E*_α_	Activation energy
*Q*	Heat flux
Z_α_/Z_0.5_	Normalised generalised conversion function
