# Supplementary material for: A Comprehensive Kinetic Study on the Enhanced Thermal Stability of Silica Xerogels with the Addition of Organochlorinated Substituents
Source: Gels. 2025 Dec 19;12(1):2. doi: 10.3390/gels12010002 (PMC12840895; doi:10.3390/gels12010002)
Supplement: Supplementary file 1 [file gels-12-00002-s001.zip › gels-3986245-supplementary.pdf]

Supplementary Material

Enhanced Thermal Stability of Silica Xerogel with the Addition of Organochlorinated Ligands

Beatriz Rosales-Reina [1], Guillermo Cruz-Quesada [2], Pablo Pujol [3], Santiago Reinoso [1], César Elosúa [4], Gurutze Arzamendi [1,*], María Victoria López-Ramón [2], Julián J. Garrido [1,*]

1 Institute for Advanced Materials and Mathematics (INAMAT[2]), Departamento de Ciencias, Universidad Pública de Navarra (UPNA), Campus de Arrosadía, 31006 Pamplona, Spain; beatriz.rosales@unavarra.es (B.R.-R.); santiago.reinoso@unavarra.es (S.R.); garzamendi@unavarra.es (G.A.); j.garrido@unavarra.es (J.J.G.)

2 Departamento de Química Inorgánica y Orgánica; Facultad de Ciencias Experimentales, Universidad de Jaén, 23071 Jaén, Spain; gcruz@ujaen.es (G.C.-Q.); mvlro@ujaen.es (M.V.L-R.)

3 Unidad Científico Técnica de Apoyo a la Investigación (UCTAI), Universidad Pública de Navarra (UPNA), Campus de Arrosadía, 31006 Pamplona, Spain; pablo.pujol@unavarra.es (P.P.)

4 Institute of Smart Cities (ISC), Departamento de Ingeniería Eléctrica, Electrónica y de Comunicación, Universidad Pública de Navarra (UPNA), Campus de Arrosadía, 31006 Pamplona, Spain; cesar.elosua@unavarra.es (C.E.)

* Correspondence: garzamendi@unavarra.es (G.A.); j.garrido@unavarra.es (J.J.G.) Tel.: (+34 948 16 9601 (J.J.G))

Table of Contents

Figure S1. FTIR obtained from ref at the maximum value of GC-MS.[1] 3
Figure S2. Thermal evolution of the relative abundance of the organic fragments detected in the vapours from the pyrolysis or organochlorinated xerogels at different decomposition stages *[29]* 4
Figure S3. Linearity of the FWO method for the thermal decomposition of ClMTEOS, ClETEOS, and ClPhTEOS using ln(β) vs 1/T plots within the α = 0.05–0.95 range with increments of 0.025 5
Figure S4. Dependence of the molar enthalpy change with α for the TEOS reference and the four organochlorinated ClRTEOS xerogels 5
Figure S5. Dependence of the Gibbs energy change with α for the TEOS reference and the four organochlorinated ClRTEOS xerogels 6
Figure S6. Criado master plots for the three decomposition stages of the TEOS reference and the four organochlorinated ClRTEOS materials using the different Pn, An, Dn, and Rn models collected in Table S1 7
Tabla S1. Values of the mass loss for each heating rate at each interval of temperature for studied materials 8
Table S2. Fitting performance of various kinetic models with different values of $f(\alpha)$ and $g(\alpha)$ 9

The identification of the different species was carried out in a previous work [1] at a heating rate of β = 40 K·min⁻¹, in order to ensure their maximum concentration, as discussed in that study. Figure S1 shows the FT-IR spectrum at the point of maximum thermal decomposition, and Figure S2 shows the GC-MS spectrum, where the peaks were labelled with numbers corresponding to fragments with their associated mass-to-charge (m/z) ratios’

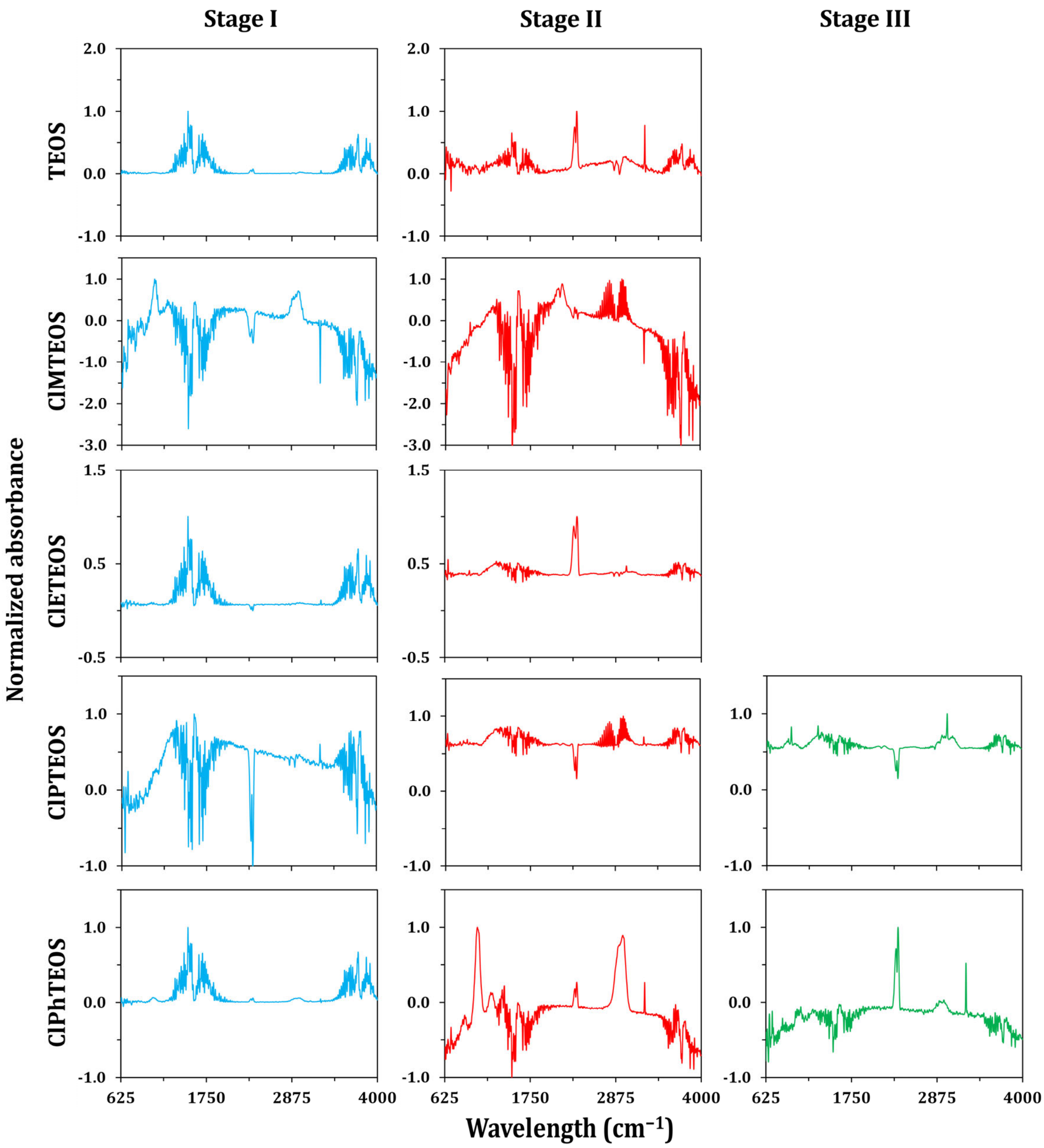

Figure S1. FTIR obtained from ref at the maximum value of GC-MS.

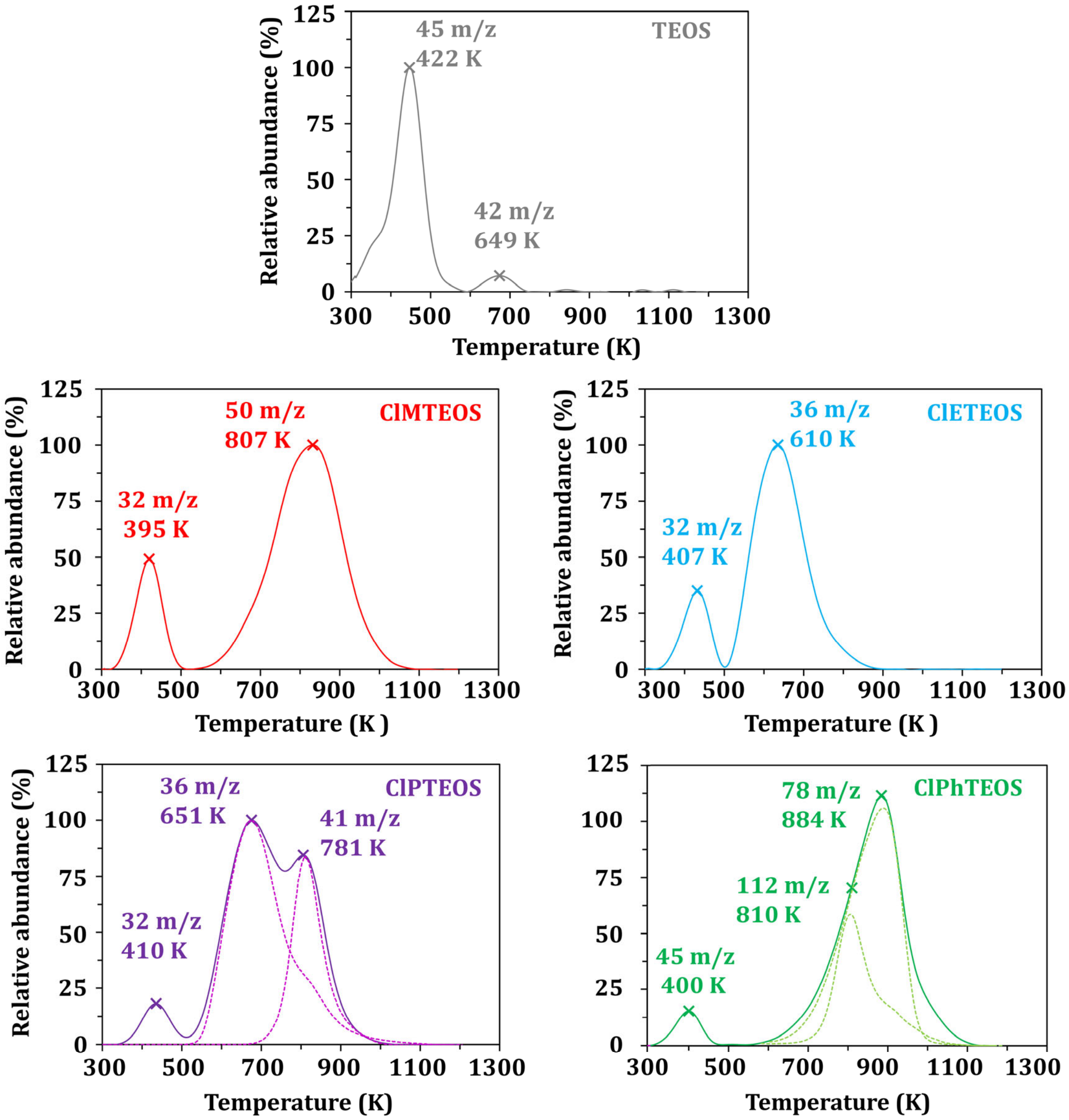

Figure S2. Thermal evolution of the relative abundance of the organic fragments detected in the vapours from the pyrolysis or organochlorinated xerogels at different decomposition stages [1]

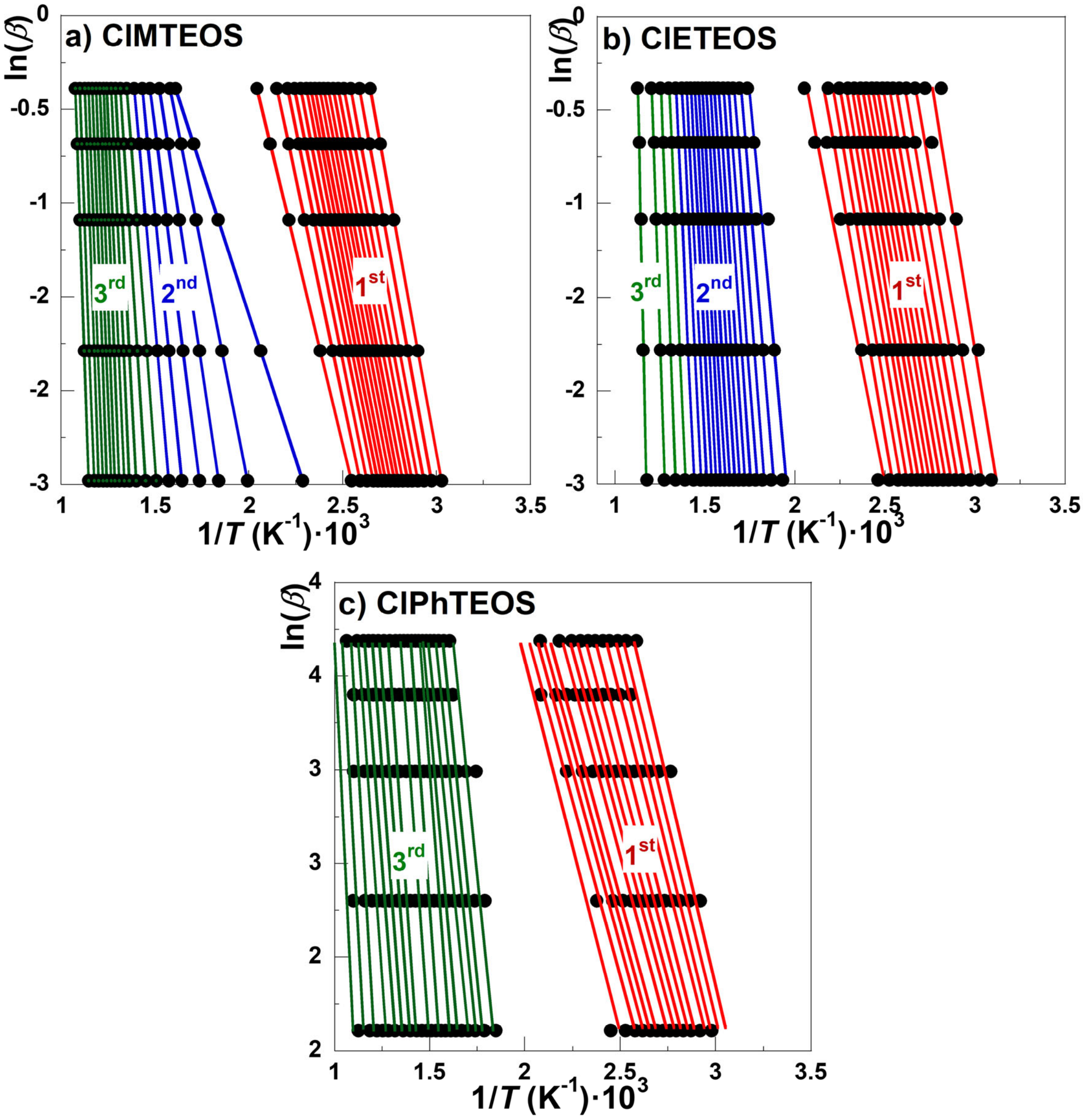

Figure S3. Linearity of the FWO method for the thermal decomposition of ClMTEOS, ClETEOS, and ClPhTEOS using ln(β) vs 1/T plots within the α = 0.05–0.95 range with increments of 0.025.

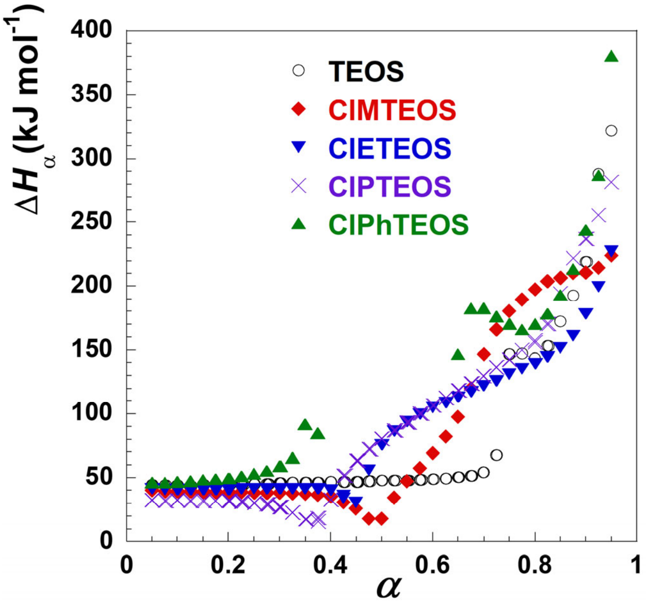

Figure S4. Dependence of the molar enthalpy change with α for the TEOS reference and the four organochlorinated ClRTEOS xerogels.

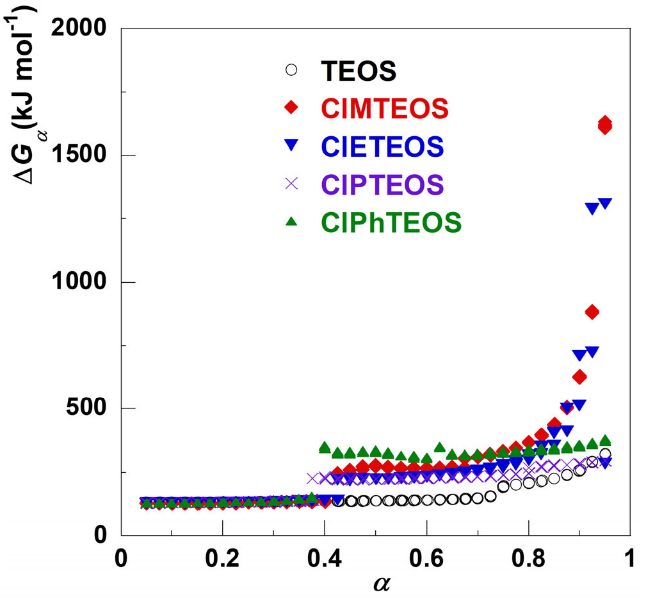

Figure S5. Dependence of the Gibbs energy change with α for the TEOS reference and the four organochlorinated ClRTEOS xerogels.

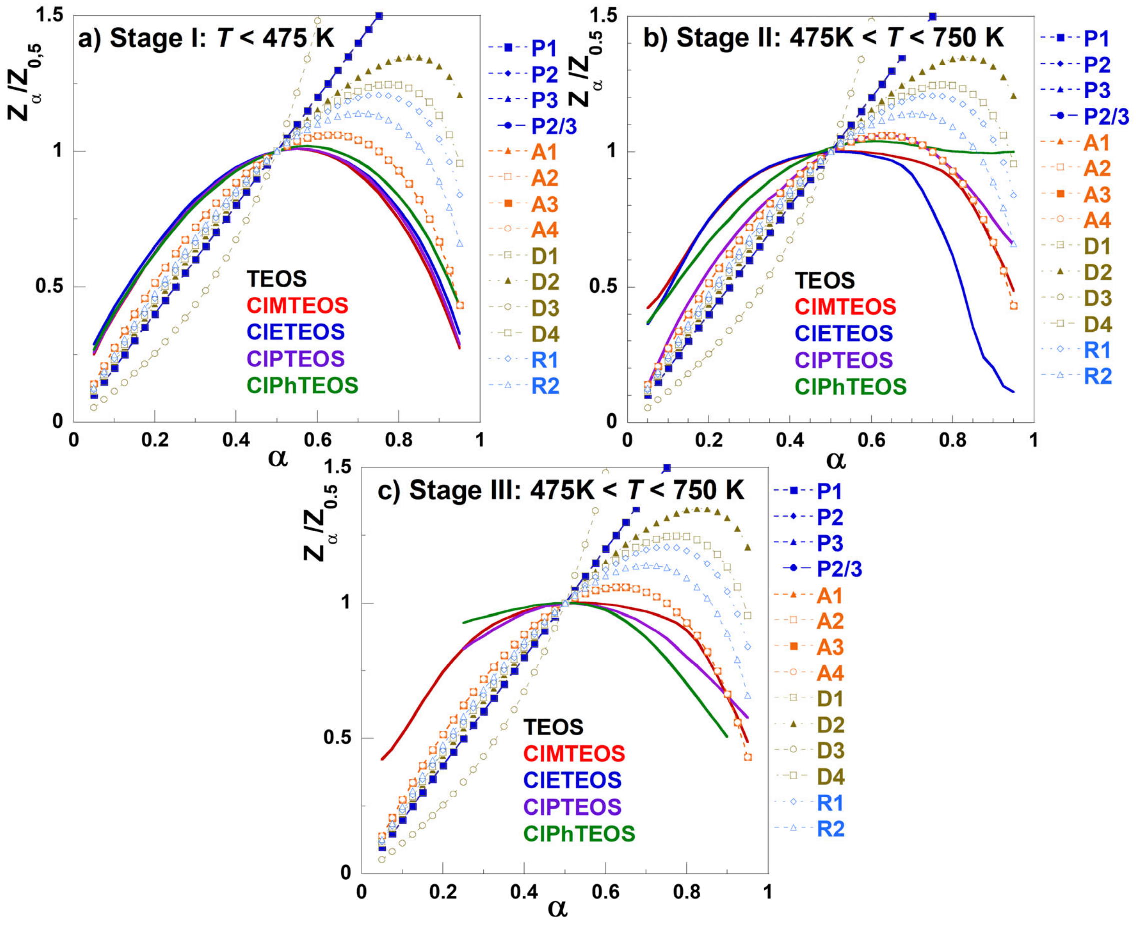

Figure S6. Criado master plots for the three decomposition stages of the TEOS reference and the four organochlorinated ClRTEOS materials using the different Pn, An, Dn, and Rn. models collected in Table S1.

Table S1. Values of the mass loss for each heating rate at each interval of temperature for studied materials.

XG	β (K min^{-1})	First interval temperature (K)	m_{loss1} (%)	Second interval Temperature (K)	m_{loss2} (%)	Third interval temperature (K)	m_{loss3} (%)
TEOS	5	303.0–467.1	14.4	467.1–751.8	4.6		
	10	303.0–469.6	14.7	469.6–769.9	4.9		
	20	303.0–495.7	14.2	495.7–773.0	4.4		
	30	303.0–517.6	15.2	517.6–789.8	4.5		
	40	303.0–520.1	20.9	520.1–801.1	6.8		
ClMTEOS	5	303.0–423.6	10.1	679.8–923.4	12.5		
	10	303.0–481.4	11.4	481.4–971.5	12.1		
	20	303.0–518.1	10.1	518.1–960.35	12.1		
	30	303.0–517.5	9.5	517.5–1003.6	11.9		
	40	303.0–481.4	8.6	481.4–1009.2	12.0		
ClETEOS	5	303.0–481.4	8.9	481.4–710.1	9.4		
	10	303.0–440.6	8.8	440.6–724.7	9.2		
	20	303.0–488.2	9.1	488.2–744.1	9.3		
	30	303.0–509.6	8.4	509.6–757.2	9.1		
	40	303.0–495.9	7.5	495.9–764.7	9.2		
ClPTEOS	5	303.0–444.8	8.2	444.8–748.8	10.6	748.8–861.0	3.3
	10	303.0–480.7	8.2	480.7–764.7	10.4	764.7–880.3	3.3
	20	303.0–503.5	8.3	311.4–780.9	10.7	780.9–889.6	3.2
	30	303.0–521.8	6.7	521.8–790.4	10.7	790.4–894.1	3.1
	40	303.0–524.0	6.6	524.0–799.1	10.5	799.1–912.7	3.2
ClPhTEOS	5	303.0–414.6	6.9	414.6–848.6	8.7	848.6–1004.4	5.6
	10	303.0–414.6	6.7	428.9–896.6	9.1	857.3–1010.7	4.3
	20	303.0–445.1	6.8	445.1–875.9	7.9	875.9–1042.4	6.2
	30	303.0–481.1	8.4	481.1–889.1	7.9	889.1–1047.5	5.8
	40	303.0–474.0	7.2	474.0–890.0	8.0	890.0–1042.4	4.8

Table S2. Fitting performance of various kinetic models with different values of *f(α)* and *g(α)*.

Solid-state mechanism	Symbol	$f(\alpha)$	$g(\alpha)$
**Reaction order models**			
First order	F1	$1-\alpha$	$-\ln(1-\alpha)$
Second order	F2	$(1-\alpha)^2$	$(1-\alpha)^{-1}-1$
Third order	F3	$(1-\alpha)^3$	$(1/2)\,[(1-\alpha)^{-2}-1]$
**Exponential nucleation models**			
Power law	P2	$2\alpha^{1/2}$	$\alpha^{1/2}$
Power law	P3	$3\alpha^{2/3}$	$\alpha^{1/3}$
Power law	P4	$4\alpha^{3/4}$	$\alpha^{1/4}$
Power law	P2/3	$2/3\alpha^{-1/2}$	$\alpha^{3/2}$
**Random nucleation and nuclei growth models**			
Avrami-Erofeev	A1	$1/2(1-\alpha)[-\ln(1-\alpha)]^{1/3}$	$[-\ln(1-\alpha)]^{2/3}$
Avrami-Erofeev	A2	$2(1-\alpha)[-\ln(1-\alpha)]^{1/2}$	$[-\ln(1-\alpha)]^{1/2}$
Avrami-Erofeev	A3	$3(1-\alpha)[-\ln(1-\alpha)]^{2/3}$	$[-\ln(1-\alpha)]^{1/3}$
Avrami-Erofeev	A4	$4(1-\alpha)[-\ln(1-\alpha)]^{3/4}$	$[-\ln(1-\alpha)]^{1/4}$
**Diffusion models**			
One-dimensional diffusion	D1	$1/2\alpha^{-1}$	α^2
Two-dimensional diffusion (Valensi model)	D2	$[-\ln(1-\alpha)]^{-1}$	$\alpha+(1-\alpha)\ln(1-\alpha)$
Three-dimensional diffusion (Jander Moder)	D3	$3/2(1-\alpha)^{2/3}[1-(1-\alpha)^{1/3}]^{-1}$	$[1-(1-\alpha)^{1/3}]^2$
Three-dimensional diffusion (Ginstlinge-Brounshtein model)	D4	$3/2\,[(1-\alpha)^{1/3}-1]^{-1}$	$1-2/3\,\alpha-(1-\alpha)^{2/3}$
**Geometrical contraction models**			
Contracting cylinder	R2	$2(1-\alpha)^{1/2}$	$1-(1-\alpha)^{1/2}$
Contracting sphere	R3	$3(1-\alpha)^{2/3}$	$1-(1-\alpha)^{1/3}$

References

1. Rosales-Reina, B.; Cruz-Quesada, G.; Pujol, P.; Reinoso, S.; Elosúa, C.; Arzamendi, G.; López-Ramón, M.V.; Garrido, J.J. Determination of Hazardous Vapors from the Thermal Decomposition of Organochlorinated Silica Xerogels with Adsorptive Properties. *Environ. Res*. **2024**, *256*, 119247. https://doi.org/10.1016/j.envres.2024.119247.
